# Viral Proteins with PxxP and PY Motifs May Play a Role in Multiple Sclerosis

**DOI:** 10.3390/v14020281

**Published:** 2022-01-28

**Authors:** Keng-Shuo Pi, Yurou Sang, Suzana K. Straus

**Affiliations:** 1 Department of Chemistry, University of British Columbia, 2036 Main Mall, Vancouver, BC V6T 1Z1, Canada; alexpi@chem.ubc.ca (K.-S.P.); yurou.sang@alumni.ubc.ca (Y.S.); 2 Faculty of Health Sciences, Simon Fraser University, 8888 University Drive, Burnaby, BC V5A 1S6, Canada

**Keywords:** multiple sclerosis, proline-rich domains, SH3 domains, E3 ubiquitin ligases, WW domains

## Abstract

Multiple sclerosis (MS) is a debilitating disease that arises from immune system attacks to the protective myelin sheath that covers nerve fibers and ensures optimal communication between brain and body. Although the cause of MS is unknown, a number of factors, which include viruses, have been identified as increasing the risk of displaying MS symptoms. Specifically, the ubiquitous and highly prevalent Epstein–Barr virus, human herpesvirus 6, cytomegalovirus, varicella–zoster virus, and other viruses have been identified as potential triggering agents. In this review, we examine the specific role of proline-rich proteins encoded by these viruses and their potential role in MS at a molecular level.

## 1. Multiple Sclerosis

MS is an autoimmune disease in the central nervous system (CNS) [1]. The disease typically arises in young adults, with most common symptoms consisting of dysesthesia, fatigue, spasticity, numbness, walking difficulties, weakness, and cognitive changes. The disease occurs in four different stages. Most MS patients start with a course of relapsing-remitting MS (RRMS), during which inflammation in the CNS is temporary. More than 80–90% of patients experience RRMS with neurological issues separated by complete or incomplete remission [2,3]. RRMS can then lead to secondary progressive MS (SPMS). This is often accompanied by gradual neurologic deterioration not associated with acute attacks [4]. Finally, some patients experience primary-progressive MS (PPMS), where the disease progresses slowly and steadily, without any remission periods or progressive-relapsing MS (PRMS), which is like PPMS but with periods of relapse in between. Since 2013, however, clinical course descriptors of MS do not include PRMS [5,6,7]. A useful graphic which explains the various stages of MS can be found in the excellent review of Tarlinton et al. [8]. Although there have been immense strides made in the last decade to slow some of the negative effects of MS [9,10], there is still no cure.

The mechanism that gives rise to MS is generally thought to involve gradual demyelination and loss of oligodendrocytes due to an autoimmune response generated against self-antigens [11,12]. Loss of functional oligodendrocytes, which are responsible for effective signal transduction between neurons through maintaining healthy myelin sheath, result in clinical presentations of plaques or lesions in the optic nerve, brain stem, basal ganglia, and spinal cord white matter [13]. The causes that lead to demyelination and loss of oligodendrocytes are not fully understood, but generally the following risk factors have been identified [14,15,16,17]: (i) immune system; (ii) genetics; (iii) environment; and (iv) infection. In the case of the immune system, B-cells and T-cells have been identified to play a role. Activated B-cells travel to the CNS, giving rise to antibodies and other proteins that contribute to CNS damage [3]. Indeed, one of the earliest evidence that antibodies play a role in MS was the presence of immunoglobulins (IgG) in cerebrospinal fluid in a high proportion of MS patients. The source of these IgGs was confirmed to be clonal B-cells by transcriptomic studies [18]; however, there is no evidence for an MS-specific autoantibody, suggesting that the role of B-cells in neuroinflammation is not primarily due to antibody production [3]. This is further supported by the finding that treatments for MS patients targeting B-cells that do not produce antibodies show promise [19,20]. Many more studies have shown the implication of T-cells in MS: demyelination occurs as a result of an autoimmune attack by autoreactive T-cells [14,21,22,23]. Autoantigens, i.e., normal constituents of neuronal cells that become the target of an immune response include myelin basic protein (MBP) and proteolipid protein (PLP), as well as the less prevalent minor components of myelin sheaths, namely myelin oligodendrocyte glycoprotein (MOG) and myelin-associated glycoprotein (MAG) [24]. Genetic factors linked to MS consist of a combination of genes that are involved in immune system regulation. Initial studies pointed to the involvement of HLA alleles [25,26], but more recent work identified 200 loci linked to susceptibility to MS away from the HLA region [27,28,29]. Moreover, a number of studies suggest that genetic factors alone are insufficient and often require additional risk factors such as environmental ones, such as, e.g., an unhealthy gut microbiome [30]. Additional environmental factors include low vitamin D or low sun exposure, air pollution and exposure to organic solvents. These risk factors are moderate to weak and the connection to MS onset and progression is extremely complex and varies between population groups [31]. Finally, it has been suggested that one or more viruses may be possible triggers of MS [8,21,22,32]. This will be presented in more detail in the following section.

Overall, because the etiology of MS remains largely unknown and because it is likely to be a multifactorial disease, the factors listed above could each play a role and affect immune system development and response in individuals, resulting in MS onset and disease progression. The inadequate understanding of the pathogenesis of MS makes it difficult to identify potential target molecules needed to design new therapeutic drugs [33]. Hence, a better, i.e., molecular level, understanding of what some of the factors implicated in MS might be could provide new treatment avenues.

## 2. Viral Risk Factors

A number of viruses have been identified as risk factors for MS or as potential triggers. These include the human herpes viruses Epstein–Barr virus (EBV), human herpes virus-6 (HHV-6), varicella zoster virus (VZV) [34,35], and cytomegalovirus (CMV), as well as endogenous retroviruses (ERVs) [8]. In some cases, herpes simplex virus type 1 (HSV-1) [36] and the non-herpes virus human polyomavirus 2 or JC virus (JCV) have also been implicated [8]. Many of these viruses are ubiquitous and latent. In the following subsections, these viruses will be discussed individually.

### 2.1. Epstein–Barr Virus (EBV)

EBV, the cause of infectious mononucleosis, is found in more than 90% of the population. After infection, it lies dormant in B-cells. Studies [37,38,39,40] have shown that EBV infection is needed for MS to arise and high anti-EBV antibody titers are present before a diagnosis is even made. Moreover, MS patients show elevated CD4 and CD8 T-cell responses to EBV or impaired EBV-specific CD8 T-cell responses [41,42,43,44,45]. A phenomenon called T-cell exhaustion is proposed to be the result, i.e., a state of T-cell dysfunction that arises due to persistent active infection [46]. Although the exact mechanism by which EBV plays a role in MS is unknown, a number of possible avenues have been suggested: cross-reactivity between EBV and MBP (i.e., molecular mimicry) [47,48]; increased production of alpha-B-crystallin due to EBV [49], a small heatshock protein that plays a role in regulating neuroinflammation [50]; and finally, antibody-dependent cell-mediated cytotoxicity [46].

Although mimicry is unlikely to be the unique mechanism for linking EBV and MS [51], it offers an interesting molecular level insight into how autoimmunity may be induced. Myelin basic protein or MBP is the primary protein found in the myelin sheath, responsible for the adhesion of the cytosolic side of lipid layers. It is encoded in a larger gene called **g**ene of the **ol**igodendrocyte **li**neage or golli. The protein is produced in a variety of sizes ranging from 14 kDa to 21.5 kDa due to alternative splicing, with the classic isoform being the 18.5 kDa one [52]. When an autoimmune response is triggered, MBP acts as an autoantigen, with anti-MBP autoantibodies readily detected in the cerebrospinal fluid (CSF) of MS patients [53]. The number of these antibodies was found to increase steadily through the progressive stages of MS [54], with anti-MOG antibodies being steadily present throughout. However, extensive work suggests that the presence of anti-MOG antibodies leads to diseases distinct from MS [55].

MBP contains a seven amino acid residue sequence, rich in proline residues (Table 1). In the middle of this sequence is a threonine (Thr98), which can be phosphorylated during post-translational modification (PTM). In MS patients, less of this specific PTM has been found [56]. Interestingly, the latent membrane protein 2A (LMP2A) from EBV also has a number of proline rich segments [57,58] and sites where phosphorylation can occur [59] (Table 1). The role of these proline rich motifs and phosphorylation will be discussed in more detail in subsequent sections. LMP2A has been implicated in MS through T-cell exhaustion [60]. Support for the implication of LMP2A comes from studies by Aloisi et al. where LMP2A was detectable in the B-Cells obtained from the brains of MS patients at higher levels than the prevalent and important B-Cell marker CD19 [61,62]. Moreover, a treatment consisting of cytotoxic T-cells specific for a combination of EBV proteins, which includes LMP2A, has been applied to progressive MS patients and shown promising results [63].

In addition to LMP2A, Epstein–Barr virus nuclear antigen 1 (EBNA1), EBNA2 and EBNA3 have all been found to potentially play a role in MS [64]. EBNA1 is expressed in EBV infected cells and plays an important role in maintaining genome regulation. In addition, EBNA1 was found to be present in individuals harboring latent EBV infections through PCR analysis. Evidence for a link to MS comes from the detection of high titers of IgG to EBNA-1 [65] or through EBNA-1/MBP cross-reactivity [47]. EBNA2 functions by upregulating host gene expression and recruiting transcription activation factors [8,66,67,68,69]. Its link to MS has been established through the observation that EBNA2 interacts with vitamin D receptor (VDR), resulting in deficiency which is known as a predisposing factor in MS. EBNA2 has also been found to play a role in resting B-cells [70]. Finally, EBNA3 was also found to interact with the VDR [64]. The exact role of the latter two proteins in MS remains to be further characterized, as they are infrequently detected in MS lesions [71].

### 2.2. Human Herpes Virus Type 6 (HHV-6)

Firstly, isolated from patients with lymphoproliferative and HIV/AIDS related diseases in the 1980s, HHV-6 has been shown to have a significant impact on a wide variety of neurological diseases, including multiple sclerosis, encephalitis, epilepsy and Alzheimer’s [72,73,74,75,76,77,78,79]. Based on biological, immunological, epidemiological, and molecular properties, HHV-6 has been classified into two variants: HHV-6A and HHV-6B [80], which share more than 90% overall nucleotide sequence identity [81]. Aside from being able to modulate T-cell responses, the major contributor towards the pathogenicity of HHV-6 results from its efficacy in establishing life-long latency in the host cells [82]. Belonging in the same subfamily of *betaherpesvirinae*, HHV-6 and human cytomegalovirus (CMV) share similar latency features, including latency-associated transcriptional sequences [83,84,85].

Early reports established links between HHV-6 and MS through the observation of HHV-6 viral DNA in the brain and CSF of MS patients [86,87]. Further findings showed increased expression levels of HHV-6 in brain samples of MS patients relative to those of controls, as well as greater levels of mRNA and DNA in demyelinated plaques [88,89,90] and protein expression and viral mRNA in oligodendrocytes [72,88]. Furthermore, HHV-6 showed greater expression levels in acute lesions relative to that of chronic lesions as compared to EBV, VZV, HSV and CMV, possibly associating viral expression with earlier stages of MS lesion formation [88]. Additional studies demonstrated intranasal HHV-6 viral acquisition accelerated neuroinflammation in a nonhuman primate MS model [91].

At a molecular level, U24 from HHV-6 has been identified as possibly being implicated in MS because T-cell cross-reactivity between the proline rich region of myelin basic protein (Table 1) and the first thirteen residues in U24 was found [92,93,94]. Indeed, U24 shares a seven amino acid residue sequence identity with this important segment of MBP (Table 1) and includes both a PxxP and a PY motif. Cheng et al. showed in addition that the CD4+/CD8+ T-cell response was higher when U24 was combined with MBP or with an immunodominant peptide from EBV (which is homologous to the segment in MBP which precedes the PxxP motif) [95].

Other proteins from HHV-6 which have been implicated in MS include U94 [96,97], a protein that plays an important role in the viral life cycle. Anti-U94A immunoglobulins have been detected in serological data from MS patients [97]. Further evidence of implication arises from a study demonstrating that the expression of HHV-6 U94A impacts migration of human oligodendrocyte progenitor cells, both in vitro and in vivo [98]. These data suggest a link between latent viruses and MS. Interestingly, U94 contains a PxxP motif.

### 2.3. Cytomegalovirus (CMV)

CMV is also a member of the *betaherpesvirinae* subfamily, and is also known as betaherpesvirus 5 (HHV-5) in humans. It is a common virus and becomes latent. Its association with multiple sclerosis is not conclusive [99,100]. A number of studies suggest a much stronger link with the viruses discussed in the sections above [100,101].

Although no specific proteins have been identified, the CMV genome encodes for two proteins, UL25 and UL42, which possess proline rich segments (Table 1) and interact with SH3 domains and WW domains, respectively [102,103,104].

### 2.4. Other Viruses

Varicella Zoster Virus (VZV) is a pathogenic human herpes virus and the cause of the primary infection known as varicella or chicken pox. As other herpes viruses, it becomes latent, primarily in neurons [8,105]. Although some studies pointed to a role for VZV in MS [106,107], other studies suggest that the link is non-existent [108]. VZV has a protein containing a PY motif (Table 1), which interacts with the E3 ubiquitin ligase ITCH [103], and other proline-rich segments.

Herpes Simplex Virus type 1 (HSV-1) is a widespread human viral pathogen and affects epithelial cells during primary infection, establishing itself latently in ganglia, the olfactory bulb, the brainstem, or the temporal cortex [36]. HSV-1 is considered to be a risk factor for MS through its ability to mediate the activity of endogenous retroviruses (ERVs) (*vide infra*). Other implications of HSV in MS come from antibody prevalence of HSV-1 and 2 in MS patients [8], though some studies call the importance of this into question [109]. UL56 from HSV-1 (Table 1) has two PY motifs, as well as PxxP motifs [103].

Approximately 8% of the human genome currently consists of a sequence that is retroviral in origin. This sequence encodes for human endogenous retroviruses or HERVs. The first report of the role HERVs may play in MS was over three decades ago, when the retrovirus multiple sclerosis-associated retroviruses (MSRV) were first detected in cell cultures [110]. More recent studies [111,112,113] suggest, however, the association of HERVs with MS comes from the activation of, e.g., HERV-H, HERV-K, and HERV-W through the presence of herpes viruses (e.g., EBV [114,115], VZV, HSV-1 and HHV-6A/B [115,116]). Interestingly the MSRV-Env protein, which is detected in MS brain lesions [117], has two PxxP motifs. Moreover, the HERV-W Env protein syncytin has the same two PxxP motifs and has been demonstrated to be present in the CNS of MS patients, as well as healthy individuals [118,119,120].

Finally, human polyomavirus 2 or JC virus (JCV) is another highly ubiquitous, latent virus, with associations to MS. It is linked to several brain diseases, particularly in immune-compromised patients [121], such as progressive multifocal leukoencephalopathy (PML) [122]. The link between JCV, PML and MS is indirect: PML was first reported after the α4 integrin monoclonal antibody natalizumab, approved for treatment of multiple sclerosis, was widely used [123].

Overall, the common themes that emerge when examining the viral link to MS are that: (i) the viruses implicated are ubiquitous, and hence highly seropositive; (ii) they are latent; and (iii) many encode for proteins with proline-rich regions. The first two points imply that it is often extremely difficult to establish causality between the virus and disease [124,125]. Indeed, it has been suggested that strict criteria be used when linking ubiquitous transmissible agents that produce lifelong infections to any neurological diseases [124]. In light of understanding at a molecular level what the viral implication in MS might be, it would also be important to better understand whether proline-rich proteins are likely to play an important role.

## 3. Proline-Rich Proteins and Their Ligands

**Figure 1:**
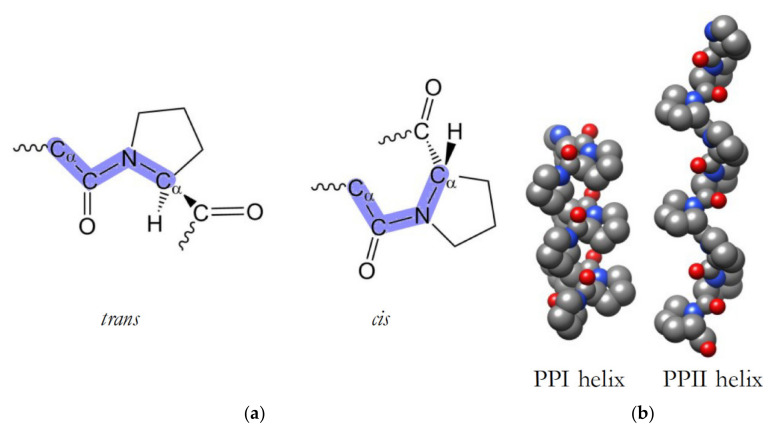
x-proline bond isomers and polyproline type I and type II helices. (**a**) *trans* and *cis* isomers of x-Pro peptide bonds, where x is any residue preceding the proline. The bonds defining the geometries are highlighted in blue. (**b**) Side views of PPI and PPII helices, comprised of nine proline residues. The helices are oriented such that the top represents the N-terminus. The figure was generated using USCF Chimera [129]. Carbon atoms are shown in grey, nitrogen in blue and oxygen in red. Hydrogen atoms are omitted for clarity.

Proline is a unique amino acid, since its side chain is covalently connected to the backbone nitrogen in the peptide bond. This has the consequence that no amide proton is available for hydrogen bonding and thus stabilizing secondary structures. In a protein or peptide, the peptide bond between proline and the previous residue in the sequence adopts two possible conformations, namely *cis* or *trans* (Figure 1a). In proteins that contain segments with multiple prolines (i.e., a proline-rich or polyproline segment), two types of helical structures can be adopted (Figure 1b): (i) a type II helix (PPII helix) is formed when the x-Pro bond is *trans*, where x represents the amino acid preceding the proline; or (ii) a less common type I helix (PPI helix) with *cis* x-Pro bonds. The PPII left-handed helix is elongated, with a structure that is maintained without stabilizing hydrogen bonds.

Proline-rich segments are typically involved in protein–protein interactions because of the distinct structure of the polyproline helix. In a PPII helix, the lack on intramolecular hydrogen bonds (mentioned above) means that the carbonyl moieties point away from the helix long axis, instead of along the axis as in an α helix. Consequently, the carbonyls are free to easily participate in forming intermolecular hydrogen bonds [126]. In addition, the size and shape of the cyclic proline side chain are often an ideal fit for a hydrophobic pocket situated on a protein surface [127,128]. Indeed, proline-rich segments are ideal binding surfaces for binding SH3 and WW domains, discussed in the following subsections.

### 3.1. PxxP Motifs Bind to Src Homology 3 (SH3) Domains

SH3 domains are non-catalytic domains found in kinases and phospholipases [130] that play a role in substrate recognition. They play crucial roles in many biological processes, such as regulation of enzymes and increasing the local concentration or altering the subcellular localization of components of signalling pathways [130]. These domains are small, consisting of about 50–70 amino acids, and ubiquitous. They adopt a structure consisting primarily of β sheets and loops. They do not possess a distinct binding pocket [131], indicating some flexibility in binding and relatively moderate affinity [130]. Indeed, a ligand containing a PxxP motif can bind to a SH3 domain in two possible orientations [132]. The presence of charged side chains, positioned either before or after the core PxxP motif can enhance the binding affinity (e.g., RxxPxxP or PxxPxR motifs) [132,133,134,135].

### 3.2. PPxY (PY) Motifs Bind to WW Domains

WW domains have two highly conserved tryptophan residues found in the sequence, usually ca. 20–22 amino acids apart. These domains adopt one of the smallest known β-stranded folds. They are typically monomeric in solution, without any associated cofactors or disulphide bonds [136]. WW domains have a large number of binding partners, resulting in signalling complexes that are implicated in different cellular activities and diseases, such as gene transcription and ubiquitination, or Liddle’s syndrome, Huntington’s, cancer, etc. [128,137,138,139,140]. WW domains have been classified into four groups, each with specific preferred proline-rich ligands:

- group 1 WW domains bind PPxY motifs;
- group 2 WW domains bind PPLP motifs;
- group 3 WW domains bind PPR motifs;
- and group 4 prefer the p(S/T)P motif, where pS or pT indicates phospho-serine or phospho-threonine, respectively [141,142].

## 4. SH3 and WW Domains Implicated in MS

**Figure 2:**
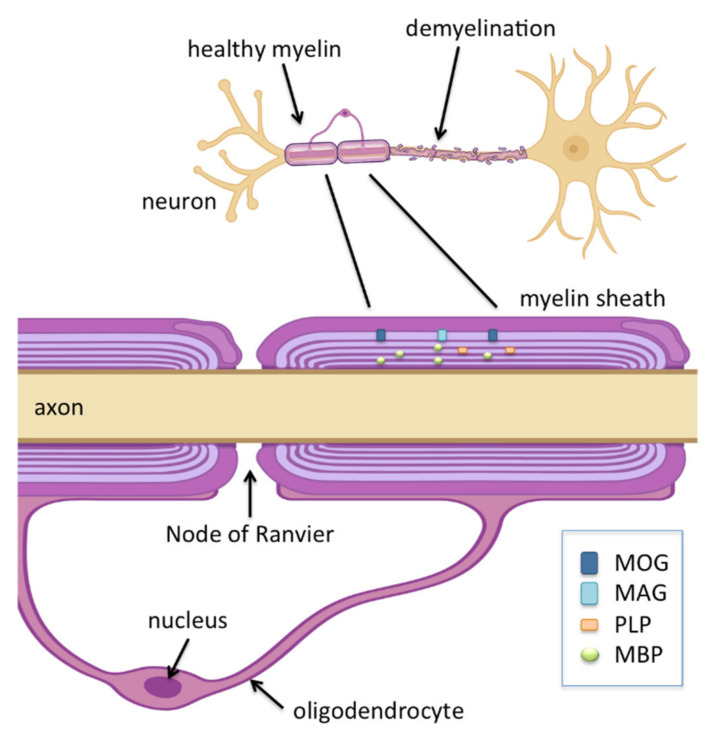
Key components of the myelin sheath, the protective layer around axons. Myelin basic protein (MBP) and proteolipid protein (PLP) are the most abundant proteins in CNS myelin. Myelin oligodendrocyte glycoprotein (MOG) and myelin-associated glycoprotein (MAG) are minor components. Oligodendrocytes, large glial cells found in the CNS, play an important role in myelin biogenesis. Oligodendrocyte precursor cells (not shown), which comprise a large population of proliferating cells in mature CNS, play a role in remyelination.

MS is a disease that leads to degradation of the protective myelin sheath, which is held together by the key components MBP and PLP, along with minor components MAG and MOG (Figure 2). These proteins or others that regulate the level of these proteins interact with SH3 and WW domains, in processes that affect myelination.

### 4.1. MBP/Fyn-SH3 Interactions

One of the important binding partners of MBP is Fyn tyrosine kinase, a protein with a unique role in myelination [143,144] and oligodendrocyte differentiation [145,146]. Expression levels of Fyn were found to be up-regulated during the initial stages of myelination [147]. Fyn is activated through the stimulation of cell surface receptors such as MAG. Fyn also promotes gene transcription of MBP, with the aid of other kinases and transcription factors. This stimulation of Fyn is stage and isoform specific [147,148]. In in vitro cellular experiments, Fyn and MBP were found to colocalize in oligodendrocytic cell lines. The interaction between Fyn and MBP occurs via the SH3 domain of Fyn tyrosine kinase (Fyn-SH3) and the PxxP motif in MBP [149,150,151]. When MBP is phosphorylated at Thr98, the affinity between MBP and Fyn-SH3 is lowered. This is due to the electrostatic repulsion between the negative surface charge on Fyn-SH3 domain, and the negatively charged ligand segment of PxxP in MBP [151].

Given the importance of MBP to maintaining the structure of myelin and given its regulation by essential signalling kinases such as Fyn, a depletion of Fyn would have a direct impact on the health of the myelin sheath and could lead to MS. Indeed, a number of studies have shown that a disruption in Fyn signalling pathways [152] can contribute to the development of neurodegenerative diseases such as Alzheimer’s and MS [153,154,155]. It is conceivable that this depletion in Fyn levels could arise through the interaction of Fyn-SH3 with viral proteins, such as LMP2A from EBV [156] and U24 from HHV-6A [157], which both contain one or more PxxP motifs (Table 1). Although the binding affinity between Fyn-SH3 and U24 from HHV-6A was found to be weaker than that between MBP and Fyn-SH3 [158], the suggestion that a number of viruses that potentiate each other may be collectively involved in MS, e.g., HHV-6A, HHV-6B, Epstein–Barr virus (EBV), Varicella Zoster Virus (VZV) [159], may provide the high concentration of PxxP motifs required to deplete Fyn.

### 4.2. E3 Ubiquitin Ligases and Oligodendrocytes

Oligodendrocytes (Figure 2) play an important role in myelination and, as mentioned above, loss of functional oligodendroglial cells leads to MS [13]. Oligodendrocytes respond to neuronal signals by adjusting the relative levels of proteolipid protein (PLP), using endocytosis or exocytosis. Endosomes are key transport vehicles that are important in the endocytic pathway. It has been suggested that during myelin biogenesis, myelin proteins and lipids associate into preformed myelin elements and are transported through the secretory pathway. Once located at the plasma membrane, these preformed myelin elements coalesce to form the myelin sheath [152].

Neural precursor cell (NPC) expressed developmentally down-regulated protein 4 (Nedd4) is an E3 ubiquitin ligase that plays two important roles in oligodendrocytes [160]: (i) it is needed to regulate expression during oligodendrocyte development; and (ii) it is required for proper remyelination to occur in the case of white matter injury in MS patients. Nedd4 and its family member Nedd4L (Nedd4-like) regulate sodium ion channels, growth factor receptors, endocytic adaptors and other proteins [161]. Regulation occurs through the many WW domains present in Nedd4 or Nedd4L and the PY motifs present on the target.

Recent studies have shown that Nedd4 and Nedd4L activity can be hijacked by viral proteins. Specifically, HSV, human CMV, and EBV have Nedd4-binding proteins: UL56, UL42, and LMP2A, respectively [103]. The LMP2A/Nedd4 interaction via the PY motifs (Table 1) initiate ubiquitination-dependent proteasomal degradation of targeted cellular proteins and consequently allows EBV to become latent [156]. Likewise, HHV-6A, -6B, and -7 all encode for the protein U24, which has a PY motif (Table 1) [103,162]. Indeed, it has been shown that U24 from HHV-6A and -7 bind hNedd4L-WW3* preferentially, with a dissociation constant of 6.3 ± 0.3 and 1.22 ± 0.01 µM, respectively. This is the same WW domain that interacts strongly with LMP2A [57]. Upon phosphorylation of the threonine in U24 from HHV-6A, binding becomes even stronger (K_d_ = 758 ± 27 nM). This strong interaction may deplete Nedd4 and hence have an impact on oligodendrocyte function, but this remains to be shown experimentally. At present time, the exact role of phosphorylation of U24 is also unclear, except for the fact that it occurs in an analogous region in MBP and LMP2A (Table 1).

## 5. Phosphorylation

MBP has a number of functions: from adhering to oligodendrocytes and the cytosolic surfaces of the multilayered myelin sheath; to interacting with the cytoskeleton in oligodendrocytes, in cytosolic inclusions in myelin, and in compact myelin, via an association between MBP, actin, and tubulin [163]. Many of these functions are modulated by PTMs [52,143,164,165], with the end result typically being a decrease in the highly positive charge of MBP. The most abundant and least modified form of MBP in healthy adult myelin contains +19 charges at neutral pH. In the case of phosphorylation in particular, this PTM was found to decrease the ability of MBP to polymerize actin and to bundle actin filaments, without however affecting the dissociation constant of the MBP-actin complex [163]. Phosphorylation of MBP also leads to a reduced interaction with Fyn [151]. Finally, MBP phosphorylation decreases the interaction between MBP and lipids, making it difficult to organize lipids into the multilayers found in the myelin sheath [166].

LMP2A from EBV plays important roles in vivo for viral replication and persistency. It has an N-terminal domain, which possesses 2 PY motifs (Table 1) and a number of tyrosine residues that can be phosphorylated. Specifically, the phosphorylation of tyrosine enables interactions with Lyn and Syk kinases. As a result of this activation, Lyn and Syk go on to initiate activation of various pathways leading to cell proliferation, division, activation, survival, migration, and metastasis [156]. Interestingly, a number of serines in LPM2A are also phosphorylated, including S15 that is located in a PxxP motif [59,156], but the importance of this PTM to LMP2A function appears to be as of yet unknown.

Like most viral proteins, U24 is extensively post-translationally modified when expressed in eukaryotes. The size of U24 expressed in T-cells and observed on SDS-PAGE gels [167] was found to be two times larger than the expected molecular weight of 10kDa (based on amino acid sequence). The exact modifications were, however, not identified, but phosphorylation is certainly a possible PTM. Indeed, it has been shown that recombinantly expressed U24 from HHV-6A can be phosphorylated at Thr6 [12].

**Figure 3:**
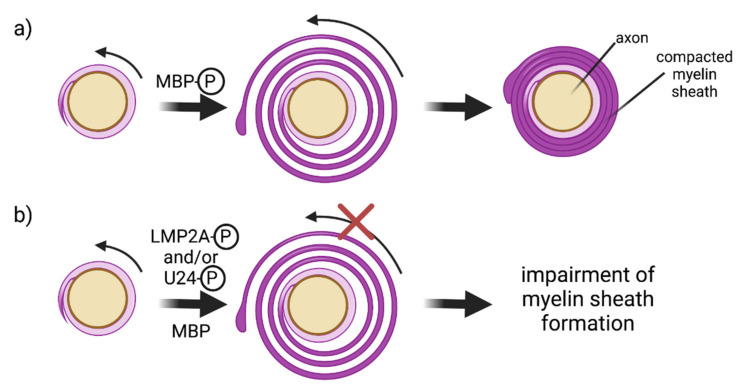
Hypothesis of how a decreased level of phosphorylation of MBP in MS patients could impact myelinogenesis in the CNS by oligodendrocytes. (**a**) Steps involved in myelinogenesis in the case of healthy patients. Adapted from Chang et al. [171]. (**b**) Hypothetical scenario in MS patients, where a decrease in MBP phosphorylation arises because of competition with viral proteins LMP2A from EBV and U24 from HHV-6. Unphosphorylated MBP should have stronger electrostatic interactions with lipids and Fyn and increase actin polymerization. As indicated in (**b**), stronger interactions between MBP and its many binding partners could impede with the dynamic processes that lead to myelin sheath formation.

If viral proteins play a role in MS, it would be important to understand what the role of phosphorylation might be. It has been shown that MBP phosphorylation occurs immediately before and during myelinogenesis (Figure 3) in the developing nervous system [166]. Moreover, Yon et al. [168] showed that a reduction in phosphorylation at Thr95 was found in MS patients, a fact corroborated by a later study by the same research group [56]. Given these observations, one possible scenario could be that viral proteins perturb MBP phosphorylation levels, thereby resulting in less phosphorylated MBP. This reduction in phosphorylated MBP could potentially in turn strengthen the interactions between MBP and lipids, between MBP and Fyn and increase the ability of MBP to polymerize actin and to bundle actin filaments. Given the dynamic nature of myelinogenesis, it is plausible that preventing the formation of the multilayers needed for myelin sheath formation as a result of strong MBP/lipid interactions (Figure 3) would be detrimental. Likewise, since actin needs to disassemble for myelin wrapping [169], increased actin polymerization linked to decreased MBP phosphorylation could impact myelin sheath formation. Finally, a strong MBP/Fyn interaction could reduce the amount of free Fyn required in several cellular processes required for oligodendrocyte maturation and myelination and for axon–glial signal transduction [170]. Detailed experiments that test these hypotheses could provide important insights.

## 6. Conclusions

As outlined here, there is some evidence of the implication of viruses in MS. Specifically, EBV and HHV-6A could perhaps play more significant roles than the other viruses that have also been identified, given the more extensive studies performed to date. As both these viruses encode proteins that possess PxxP and PY motifs and residues that can be phosphorylated (Table 1), and because of their sequence similarity/identity with MBP, it is conceivable that the proteins LMP2A and U24 might play more significant roles in the disease than any of the other proteins listed in Table 1. U24 from HHV-6A (U24-6A) in particular has been shown to mimic MBP to some extent by interacting with Fyn-SH3 [157], to affect endocytic recycling by binding hNedd4L-WW3* domains [162], and finally to be phosphorylated at Thr6 [12]. In addition, it has been shown that phosphorylation of U24-6A helps to strengthen the interaction between pU24-6A and hNedd4L-WW3* domains.

In conclusion, multiple sclerosis is a multifactorial disease. We have demonstrated here that viruses, and more particularly some of the proline-rich proteins expressed by theses viruses, may play a role in disease triggering or disease progression. Much research remains to be carried out to obtain crucial molecular level knowledge of how these proteins work, thereby ensuring that the causes of MS will be better understood in the future.

## Figures and Tables

**Table 1:** Sequence alignment of a number of key segments from proteins which are implicated in MS. Amino acids with negatively charged side-chains are indicated in blue, whereas those with positive side-chains are shown in red. The PxxP motifs are indicated with dark grey shading. The PPxY motifs are shaded in light grey and boxed. Residues that are phosphorylated are shown in bold.

Source	Protein Name	Segment Sequence															
myelin	MBP_93–107_	I	V	T	P	R	**T**	P	P	P	S	Q	G	K	G	R	
EBV	LMP2A_10–105_	G	A	G	P	P	**S**	P	G	G	D	P	D	G	D	D	G
		G	N	N	S	Q	Y	P	S	A	S	G	S	S	G	N	T
		P	T	P	P	N	D	E	E	R	E	S	N	E	E	P	P
		P	P	Y	E	D	P	Y	W	G	N	G	D	R	H	S	D
		Y	Q	P	L	G	T	Q	D	Q	S	L	Y	L	G	L	Q
		H	D	G	N	D	G	L	P	P	P	P	Y	**S**	P	R	D
HHV-6A	U24_1–15_	M	D	P	P	R	**T**	P	P	P	S	Y	S	E	V	L	
HHV-6B	U24_1–15_	M	D	R	P	R	**T**	P	P	P	S	Y	S	E	V	L	
HHV-7	U24_1–15_	M	-	T	H	E	T	P	P	P	S	Y	N	D	V	M	L
CMV	UL25_623–638_	C	R	S	P	P	P	P	L	P	P	R	D	Y	P	Q	R
	UL42_31–46_	S	T	P	P	P	P	P	P	D	C	S	P	P	P	Y	R
VZV	ORF0_37–52_	A	E	A	V	A	D	A	P	P	P	Y	R	S	R	E	S
HSV-1	UL56_15–55_	A	G	N	A	F	A	D	P	P	P	Y	D	S	L	S	G
		R	N	E	G	P	F	V	V	I	D	L	D	T	P	T	D
		P	P	P	P	Y	S	A	G								

## Data Availability

All data presented here is published.
